# Scheduled telephone support in internet-delivered cognitive behavioral therapy for depression: randomized controlled trials of two populations

**DOI:** 10.3389/fpsyg.2026.1704049

**Published:** 2026-02-04

**Authors:** Satu Pihlaja, Jari Lipsanen, Ville Ritola, Jan-Henry Stenberg, Grigori Joffe

**Affiliations:** 1 Faculty of Medicine, Doctoral Programme in Clinical Research, University of Helsinki, Helsinki, Finland; 2 Faculty of Medicine, University of Helsinki, Helsinki, Finland; 3 Department of Psychiatry, HUS Helsinki University Hospital, Helsinki, Finland

**Keywords:** adherence, depression, iCBT - Internet-based cognitive-behavior therapy, telephone support, therapist support

## Abstract

Among patients receiving internet-delivered cognitive behavioral therapy (iCBT) for depression in routine clinical practice, 101 patients were randomized to either iCBT as usual or to iCBT with add-on scheduled telephone support (STS). Depression symptoms improved in both groups. STS increased adherence, as measured by reaching mid-treatment and completing therapy. Unlike in our previously reported population at risk of dropout, STS did not enhance symptom improvement. STS significantly reduced the time in therapy compared to the at-risk population. STS enhances adherence to iCBT for depression, with an additional effect on severity only in the at-risk population.

## 1 Introduction

Although therapist-supported internet-delivered cognitive behavioral therapy (iCBT) programs for depression are effective in routine mental health care (Etzelmueller et al., 2020; Nordgreen et al., 2019; Kessler et al., 2009), treatment adherence is less than expected (Eysenbach, 2005; Gilbody et al., 2015; Webb et al., 2017). Various forms of therapist support have been explored to decrease dropout rates and enhance outcomes (Richards and Richardson, 2012). It has been suggested that some depressed patients may require intensive support, while others benefit from the typically offered asynchronous text-based support (Gilbody et al., 2015). Synchronous scheduled telephone support (STS) may improve adherence and outcomes for populations at risk of dropout in iCBT for OCD (Kenwright et al., 2005) and—as shown in our previous study—for depression (Pihlaja et al., 2020). However, it is unclear whether STS benefits unselected, not-at-risk patients (hereafter referred to as “unselected”). In this RCT, we examine whether STS is effective in enhancing adherence, improving outcomes, and desirably shortening treatment time for unselected patients undergoing iCBT for depression. Furthermore, we compare these potential effects with those previously reported for the at-risk population.

## 2 Methods

The study (registration number ISRCTN55123131) was approved by the Ethics Committee of Helsinki University Hospital (HUS) and followed the Guideline for Good Clinical Practice (ICH-GCP) and Finnish National Regulations. Study participants provided informed consent after reading the detailed description of the study.

Participants (*n* = 101) were recruited from patients receiving a seven-session iCBT program for depression provided by Helsinki University Hospital (HUS-iCBT) in routine clinical practice between 2 February 2017 and 1 November 2017 (see Table 1 for baseline demographics). They were randomized to either the add-on STS group receiving weekly therapist telephone support calls (15 min) or the control group receiving standard text-based support. Simple randomization was performed by an independent investigator, concealed from other investigators, using a computer-generated random number list. Participants were allocated in a 1:1 ratio to different groups, and they could not be blinded due to the nature of the intervention. The primary outcome was adherence to treatment, assessed by the proportion of patients reaching mid-treatment and those completing the program within 6 months. Secondary outcomes included changes in depression severity, measured by the change in the internet-administered Beck Depression Inventory (BDI) score from baseline to endpoint (or premature discontinuation), and treatment duration, measured in days over the same six-month period. The results were compared with those from our earlier, methodologically similar RCT involving depression patients with a ≥ 2-week delay between acceptance and the start of HUS-iCBT (hereafter referred to as patients or populations at risk of dropout) (For details, see Pihlaja et al., 2020). For comparisons between the two datasets, four groups were defined: patients at risk of dropout received add-on STS (group 1, *n* = 50) or HUS-iCBT as usual (group 2, control, *n* = 50), and new, unselected patients received add-on STS (group 3, *n* = 51) or iCBT as usual (group 4, control, *n* = 50). Apart from the STS intervention, all groups received the same HUS-ICBT program with text-based therapist support.

**Table 1:** Baseline characteristics.

Characteristic	STS *n* = 50 (%)	Control *n* = 50 (%)	Total *n* = 100 (%)	STS vs. control
Sex, Female *n* (%)	35 (68.6)	34 (68.0)	69 (68.3)	χ^2^ (1) = 0.01, *p* = 1.00
Referred from *n* (%)				χ^2^ (5) = 11.9, *p* = 0.04*
Primary healthcare	31 (60.8)	23 (46.0)	54 (53.5)	
Private healthcare	8 (15.7)	14 (28.0)	22 (21.8)	
Occupational healthcare	2 (3.9)	10 (20.0)	12 (11.9)	
Student healthcare	4 (7.8)	1 (2.0)	5 (5.0)	
Specialized psychiatry	3 (5.9)	1 (2.0)	4 (4.0)	
Unspecified	3 (5.9)	1 (2.0)	4 (4.0)	
Marital status, *n* (%)^a^				χ^2^ (4) = 5.30, *p* = 0.26
Married	3 (15.8)	2 (11.8)	5 (13.9)	
Living together	3 (15.8)	3 (17.6)	6 (16.7)	
Not married	13 (68.4)	8 (47.0)	21 (58.3)	
Divorced	0 (0.00)	3 (17.6)	3 (8.3)	
Widowed	0 (0.00)	1 (5.9)	1 (2.8)	
Educational level, *n* (%) ^a^				χ^2^ (3) = 0.14, *p* = 0.99
Elementary school	2 (10.5)	2 (11.8)	4 (11.1)	
Secondary/vocational	10 (52.6)	9 (52.9)	19 (52.8)	
College/University Bachelor	3 (15.8)	2 (11.8)	5 (13.9)	
College/University Masters	4 (21.1)	4 (23.5)	8 (22.22)	
Employment status, *n* (%)^a^				χ^2^ (3) = 2.16, *p* = 0.54
Full-time	12 (63.2)	9 (52.9)	21 (58.3)	
Part-time	1 (5.3)	3 (17.6)	4 (11.1)	
Unemployed	5 (26.3)	3 (17.6)	8 (22.2)	
Retired	1 (5.3)	2 (11.8)	3 (8.3)	
Medication, *n* (%)^a^				χ^2^ (1) = 0.63, *p* = 0.50
None	8 (42.1)	5 (29.4)	13 (36.1)	
Present^b^	11 (57.9)	12 (70.7)	23 (63.9)	
Sick leave within 6 months^a^	7 (36.8)	9 (52.9)	16 (44.4)	χ^2^ (1) = 0.94, *p* = 0.50
Age^c^, mean (SD)	33.57 (10.95)	38.00 (12.47)	35.76 (11.88)	t (99) = 1.99, *p* = 0.06
BDI at baseline^c^, mean, (SD)	24.57 (8.83)	23.35 (10.09)	23.97 (9.44)	t (98) = 0.65, *p* = 0.52

^a^Information available for 19 patients in the STS group and for 17 patients in the control group.

^b^Anxiolytic or antidepressant.

^c^BDI: Beck Depression Inventory.

An intention-to-treat analysis using the last observation carried forward (LOCF) method was employed. ANOVA and χ^2^ tests were used to assess between-group differences in baseline characteristics and dropout rates. Group comparisons of patients reaching mid-treatment and completing treatment were performed using χ^2^ tests. The Mann–Whitney U-test was used to compare changes in BDI scores from baseline to endpoint (or last observation for dropouts) and treatment duration. Comparisons between unselected patients and those previously reported as at risk of dropout were conducted using logistic regression and analysis of variance. Based on clinical relevance, logistic regression was used to examine if there were differences in adherence: first, between unselected and at-risk populations, and second, between participants who received add-on STS and those who received standard iCBT.

## 3 Results

### 3.1 Unselected population

At baseline, no differences in depression severity were found between the unselected STS and control groups (shown in Table 1). In group 3 (STS intervention), 45 participants (88%) reached mid-treatment and 37 participants (73%) completed the program (vs. 36 (72%) and 20 (40%), respectively, in group 4, control). Add-on STS increased the likelihood of reaching both mid-treatment (χ^2^ (1) = 4.19, *p* = 0.035) and endpoint (χ^2^ (1) = 10.88, *p* < 0.001) (see Table 2).

**Table 2:** Adherence, depression, and time in treatment.

Measure	Add-on STS^a^		iCBT as usual (control)		Significance	
	At-risk (Group 1)	Unselected (Group 3)	At-risk (Group 2)	Unselected (Group 4)	At-risk	Unselected
Reached mid-treatment *n* = (%)	29 (58%)	45 (88%)	18 (36%)	36 (72%)	*χ*^2^ (1) = 4.86, *p* = 0.045*	*χ^2^* (1) = 4.19, *p* = 0.035*
Completed the program *n* = (%)	12 (24%)	37 (73%)	3 (6%)	20 (40%)	*χ^2^* (1) = 6.35, *p* = 0.023*	*χ^2^* (1) = 10.88, *p <* 0.001*
Change from baseline, BDI^b^ scores, LOCF^c^					*U* = 1455.5, *p* = 0.049*	*U* = 1019.5, *p* = 0.110
Mean (SD)	3.63 (5.94)	6.73 (6.59)	1.06 (4.82)	5.0 (7.62)		
Median (25;75)^d^	0 (0.0;8.5)	5 (1.0;13)	0 (0.0;1.0)	4 (0.0;10.5)		
Time in treatment (days)					*U* = 1190.5, *p* = 0.667	*U* = 913.0, *p* = 0.014*
Mean (SD)	136.61 (52.18)	89.63 (52.09)	141.36 (48.08)	118.66 (53.92)		
Median (25;75)^d^	166.95 (96.27;183.00)	69 (47.0;124.0)	161.5 (100.25;183.00)	120.5 (72.5;182.0)		

^a^STS: Scheduled telephone support.

_b_BDI: Beck Depression Inventory.

^c^LOCF: Last observation carried forward.

^d^25th and 75th percentiles. *Statistically significant.

BDI scores decreased slightly more in the intervention group (group 3) than in the control group (6.73 vs. 5.0 points, respectively), but this difference was not statistically significant (U (1) = 1019.5, *p* = 0.110). The average time in treatment was shorter for group 3 (STS; mean 89.63 days vs. 118.66 days in group 4, control; U = 913.0, *p* = 0.014).

### 3.2 Comparisons between the unselected and at-risk populations

#### 3.2.1 Adherence

Logistic regression analysis showed that it was more likely for unselected STS patients (group 3) to reach mid-treatment (B = 1.520, *p* < 0.001) and complete the treatment program (B = 0.2346, *p* < 0.001) compared to at-risk patients (group 1). The effect of STS on adherence did not differ between the two populations (groups 1 and 3 combined vs. groups 2 and 4 combined), with B = 0.172, *p* = 0.799 for mid-treatment and B = 0.222, *p* = 783 for treatment completion.

#### 3.2.2 Depression severity

Analysis of variance revealed a larger change in depression severity among the unselected population (groups 3 and 4 combined, mean BDI change 5.517) compared to the at-risk population (groups 1 and 2 combined, mean BDI change 1.317), *F* (1) = 15.279, *p* < 0.001. When STS and control groups were compared in both populations combined (groups 1 and 3 combined vs. groups 2 and 4 combined), the mean BDI score change was significantly greater in the STS group (4.696) than in the control group (2.847), *F* (1) = 5.706, *p* = 0.018.

#### 3.2.3 Time in treatment

Analysis of variance indicated that treatment time was significantly shorter for the unselected population (groups 3 and 4 combined) compared to the at-risk population (groups 1 and 2 combined), F (1) = 22.890, *p* < 0.001. Moreover, it was significantly shorter for the STS intervention group (groups 1 and 3) compared to the control group (groups 2 and 4), F (1) = 5.382, *p* = 0.021.

## 4 Discussion

As compared to standard text-based therapist support, in this RCT, the STS intervention improved adherence and shortened the time spent in iCBT for unselected depressed patients, as it did in our previous study with patients identified as being at risk of dropout (Pihlaja et al., 2020). Unlike in at-risk patients, STS did not significantly improve the effect of HUS-iCBT on depressive symptoms in the unselected population. For improving symptoms, standard text-based therapist support seems adequate for most patients receiving iCBT for depression. Since STS is more resource-intensive, it should be reserved for patients at risk of dropout.

Identifying predictors of attrition is crucial, as a meta-analysis found that up to 57% of patients drop out of iCBT (Richards and Richardson, 2012), and even 82% in the context of routine clinical care(Gilbody et al., 2015). In an earlier study (Pihlaja et al., 2020), we notionally defined a delay of ≥2 weeks between acceptance and the start of therapy as a predictor of dropout. This predictor seems to be indirectly supported by our current results, since our previous population (those with a delay of ≥2 weeks) demonstrated a substantially higher dropout rate compared to the current unselected population. Patients at risk of dropout also benefited more from the STS intervention than the current unselected patients.

Due to the relatively small sample size, statistical power was insufficient for identifying subgroups for optimal therapist resource allocation. Further research is needed to explore and uncover risk factors for dropout, where STS may offer the benefit of targeting individual needs. A ≥ 2-week delay before the start of iCBT seems to be a valid predictor of dropout, but more research is needed to uncover other predictors and risk factors. The efficacy of STS in iCBT for other disorders is yet to be explored. Since add-on STS is more resource-intensive than standard iCBT, its cost-effectiveness should also be thoroughly evaluated to inform managerial decision-making.

## Data Availability

The raw data supporting the conclusions of this article will be made available by the authors, without undue reservation.
